# Effect of Pre-Existing Sarcopenia on Oncological Outcomes for Oral Cavity Squamous Cell Carcinoma Undergoing Curative Surgery: A Propensity Score-Matched, Nationwide, Population-Based Cohort Study

**DOI:** 10.3390/cancers14133246

**Published:** 2022-07-01

**Authors:** Yu-Hsiang Tsai, Wan-Ming Chen, Ming-Chih Chen, Ben-Chang Shia, Szu-Yuan Wu, Chun-Chi Huang

**Affiliations:** 1 Department of Otorhinolaryngology, Lo-Hsu Medical Foundation, Lotung Poh-Ai Hospital, Yilan 265, Taiwan; b101098034@tmu.edu.tw; 2 Graduate Institute of Business Administration, College of Management, Fu Jen Catholic University, Taipei 242062, Taiwan; daisywanmingchen@gmail.com (W.-M.C.); 081438@mail.fju.edu.tw (M.-C.C.); 025674@mail.fju.edu.tw (B.-C.S.); 3 Artificial Intelligence Development Center, Fu Jen Catholic University, Taipei 242062, Taiwan; 4 Department of Food Nutrition and Health Biotechnology, College of Medical and Health Science, Asia University, Taichung 41354, Taiwan; 5 Division of Radiation Oncology, Lo-Hsu Medical Foundation, Lotung Poh-Ai Hospital, Yilan 265, Taiwan; 6 Big Data Center, Lo-Hsu Medical Foundation, Lotung Poh-Ai Hospital, Yilan 265, Taiwan; 7 Department of Healthcare Administration, College of Medical and Health Science, Asia University, Taichung 41354, Taiwan; 8 Cancer Center, Lo-Hsu Medical Foundation, Lotung Poh-Ai Hospital, Yilan 265, Taiwan; 9 Centers for Regional Anesthesia and Pain Medicine, Taipei Municipal Wan Fang Hospital, Taipei Medical University, Taipei 116081, Taiwan; 10 Department of Management, College of Management, Fo Guang University, Yilan 262307, Taiwan

**Keywords:** sarcopenia, nonsarcopenia, OCSCC, survival, prognosis

## Abstract

**Simple Summary:**

Although sarcopenia during cancer diagnosis is an independent prognostic factor for poor overall survival in patients with various cancers, whether pre-existing sarcopenia is an independent risk factor for oral cavity squamous cell carcinoma (OCSCC) remains unclear. Therefore, we conducted a head-to-head propensity score matching (PSM) study to estimate the oncological outcomes of pre-existing sarcopenia in patients with OCSCC undergoing curative surgery. Both univariate and multivariate Cox regression analyses indicated that pre-existing sarcopenia was associated with poor survival than nonsarcopenia. Old age, male sex, advanced pT, advanced pN, differentiation grade II–III, margin-positive cancer, lymphovascular invasion, and CCI ≥ 1 were significant poor prognostic factors for survival in the patients with OCSCC undergoing curative surgery.

**Abstract:**

Purpose: The effect of pre-existing sarcopenia on patients with oral cavity squamous cell carcinoma (OCSCC) remains unknown. Therefore, we designed a propensity score-matched population-based cohort study to compare the oncological outcomes of patients with OCSCC undergoing curative surgery with and without sarcopenia. Patients and Methods: We included patients with OCSCC undergoing curative surgery and categorized them into two groups according to the presence or absence of pre-existing sarcopenia. Patients in both the groups were matched at a ratio of 2:1. Results: The matching process yielded 16,294 patients (10,855 and 5439 without and with pre-existing sarcopenia, respectively). In multivariate Cox regression analyses, the adjusted hazard ratio (aHR, 95% confidence interval [CI]) of all-cause mortality for OCSCC with and without pre-existing sarcopenia was 1.15 (1.11–1.21, *p* < 0.0001). Furthermore, the aHRs (95% CIs) of locoregional recurrence and distant metastasis for OCSCC with and without pre-existing sarcopenia were 1.07 (1.03–1.18, *p* = 0.0020) and 1.07 (1.03–1.20, *p* = 0.0148), respectively. Conclusions: Pre-existing sarcopenia might be a significant poor prognostic factor for overall survival, locoregional recurrence, and distant metastasis for patients with OCSCC undergoing curative surgery. In susceptible patients at a risk of OCSCC, sarcopenia prevention measures should be encouraged, such as exercise and early nutrition intervention.

## 1. Introduction

Head and neck cancer (HNC) is the third most common cancer and the fifth leading cause of cancer deaths in men in Taiwan [1] because of betel nut chewing, cigarette smoking, and alcohol use [2,3,4,5,6,7,8,9,10]. The median age of patients with HNC in Taiwan is 55 years, indicating that they are an economically active population [1,2,3,4,5,6,7,8,9,10]; thus, improving their survival is essential. In Taiwan, the oral cavity squamous cell carcinoma (OCSCC) subtype accounts for more than 80% of HNC, whereas in Western countries, most HNCs are oropharyngeal cancers [2,3,4,5,6,7,8,9,10]. This difference is likely due to the habit of betel nut chewing in Taiwan [8,9,10]. Moreover, there are 377,713 new cases and 177,757 new deaths per year for oral cancer in the world based on the last updated GLOBOCAN (IARC, WHO) report in 2020 [11]. Despite advancements in therapeutics [8,9,10], the survival rate of HNC in Taiwan has remained dismal [1]. From the perspective of preventive medicine, if a prognostic factor for survival in patients with OCSCC can be corrected before cancer diagnosis, the factor should be screened and corrected for improving survival in OCSCC.

Sarcopenia, characterized by the loss of muscle mass, strength, and performance [12,13,14], can occur not only in overweight and underweight individuals but also in those with normal weight [15]. Unlike cachexia, sarcopenia does not require the presence of an underlying illness [16]. In addition, although most people with cachexia are sarcopenic, most individuals with sarcopenia are not considered cachectic [16]. Sarcopenia is associated with increased functional impairment, disability, fall, and mortality rates [17]. The causes of sarcopenia are multifactorial and include disuse, endocrine function alteration, chronic diseases, inflammation, insulin resistance, and nutritional deficiencies [14]. Therefore, sarcopenia and cancer cachexia-related sarcopenia are distinct conditions. Pre-existing sarcopenia can be prevented, whereas cancer-related sarcopenia cannot be prevented but can be treated.

Sarcopenia is associated with increased mortality for most cancers, except hormone-related cancers (endometrial, breast, ovarian, and prostate cancers) and hematopoietic cancers [18,19,20,21], thus making it a major prognostic factor for poor overall survival and mortality in patients with cancer [18,19,20,21]. Sarcopenia-related cancer mortality might be a consequence of treatment-related toxicity [22,23]. However, whether pre-existing sarcopenia is an independent risk factor for different cancers, including OCSCC, remains unclear. A propensity score matching (PSM)-based design can resolve this issue by maintaining balance among the confounding factors of the case and control groups—all in the absence of bias [24,25,26]. Moreover, PSM is currently the recommended standard tool for estimating the effects of covariates in studies where any potential bias may exist [24,25,26]. Therefore, we conducted a head-to-head PSM study to estimate the oncological outcomes of pre-existing sarcopenia in patients with OCSCC undergoing curative surgery.

## 2. Patients and Methods

### 2.1. Study Population

We selected patients with OCSCC who had undergone curative surgery—tumor resection and neck dissection—between 1 January 2007 and 31 December 2017 from the Taiwan Cancer Registry Database (TCRD). The follow-up period was from the index date (i.e., date of surgery) to 31 December 2018. The types and indications of neck dissection were as follows: supraomohyoid neck dissection for clinically N0 tumors [27], modified neck dissection for ipsilateral clinically positive nodes [28], and bilateral neck dissection for contralateral metastases or tumors cross the midline [29]. Adjuvant treatments indicated for patients with OCSCC were based on the National Comprehensive Cancer Network (NCCN) guidelines and patients’ tolerance [30]. The TCRD contains detailed cancer-related data of patients, including the clinical stage, cigarette smoking habit, treatment modalities, pathologic data, and grade of differentiation [5,8,9,10,31]. The study protocols were reviewed and approved by the Institutional Review Board of Tzu-Chi Medical Foundation (IRB109-015-B).

The diagnoses of the enrolled patients were confirmed after reviewing their pathological data, and patients who were newly diagnosed as having OCSCC were confirmed to have no other cancers or distant metastasis (DM). All patients with OCSCC underwent curative-intent surgery. The inclusion criteria were as follows: being aged ≥20 years, having a diagnosis of pathologic stage I–IVB OCSCC without metastasis according to the American Joint Committee on Cancer criteria (AJCC, 7th edition), and undergoing tumor resection and neck dissection. Patients were excluded if they had a history of other cancers before the index date, an unknown pathological stage, missing sex data, unclear differentiation of tumor grade, or a nonsquamous cell carcinoma pathologic type.

### 2.2. Interventions/Exposures

Our definition of sarcopenia is according to the previous study from the Taiwan NHIRD [32]. In order to diminish the selection bias of the definition of sarcopenia, we only recorded the sarcopenia from the rehabilitation specialists, orthopedics, or family physicians. We have also added the sensitivity analysis of the recorded sarcopenia from the rehabilitation specialists, orthopedics, and family physician with/without other specialties (including endocrinology department) (Supplementary Table S2). In Taiwan, the coding of sarcopenia was based on a previous Taiwan study [33]; sarcopenia was defined as the skeletal muscle mass index (SMI) of 2 standard deviations (SDs) or more below the normal sex-specific means for young persons. Patients diagnosed as having sarcopenia after OCSCC diagnosis and those with sarcopenia diagnosed within 1 year before OCSCC diagnosis (excluding cancer treatment-related and cancer cachexia-related sarcopenia) were excluded. We also supplied the sensitivity analysis for the comparison of washout time intervals of one year and two years (Supplementary Table S1).

### 2.3. Comparisons

We categorized the patients into two groups depending on whether they had sarcopenia before OCSCC diagnosis: Group 1 (nonsarcopenic OCSCC) and Group 2 (pre-existing sarcopenic OCSCC). In addition, we estimated oncological outcomes (all-cause mortality, locoregional recurrence [LRR], and DM) associated with sarcopenia. Comorbidity was assessed using the Charlson comorbidity index (CCI) [6,34]. Only comorbidities which appeared 12 months before the index date were included and they were coded and classified according to the International Classification of Diseases, Ninth Revision, Clinical Modification (ICD-9-CM) or International Classification of Diseases, Tenth Revision, Clinical Modification (ICD-10-CM) codes at the first admission or after ˃2 appearances of a diagnostic code at outpatient visits.

### 2.4. Outcomes

The oncologic outcomes were defined as all-cause death, LRR, and DM according to the previous oncologic studies [35,36,37]. All-cause mortality was the primary endpoint in both the groups. The secondary endpoints were LRR and DM.

### 2.5. Design Setting

To reduce the effects of potential confounders when comparing all-cause mortality between patients without and with sarcopenia, we performed 2:1 PSM with a caliper of 0.2 for the following variables: age, sex, years of diagnosis, AJCC pathologic stages, pathologic tumor stages (pT), pathologic nodal stage (pN), differentiation grade, surgical margin, lymphovascular invasion (LVI), adjuvant treatments, CCI scores, cigarette smoking, alcohol use, and betel nut chewing. These variables are potential prognostic factors for all-cause mortality for patients with OCSCC undergoing curative surgery. A Cox proportional hazards model was used to regress all-cause mortality in patients with OCSCC with a robust sandwich estimator used to account for clustering within matched sets [38]. Potential confounding factors for all-cause mortality for OCSCC were controlled in the PSM (Table 1). After well-matched PSM, the actual real-world data can indicate the oncological outcomes of pre-existing sarcopenia in patients with OCSCC undergoing curative surgery.

### 2.6. Statistical Analysis

The aforementioned variables might be independent prognostic factors for all-cause mortality with residual imbalance after PSM [39,40]. Therefore, multivariate Cox regression analyses were performed to calculate hazard ratios (HRs) to determine whether pre-existing sarcopenia is an independent predictor of all-cause mortality.

After adjustment for confounders, all statistical analyses were performed using SAS version 9.4 (SAS Institute, Cary, NC, USA). In a two-tailed Wald test, *p* < 0.05 was considered significant. OS, LRR, and DM were estimated using the Kaplan–Meier method and between-group differences were compared using the stratified log-rank test (stratified according to matched sets) [41].

## 3. Results

### 3.1. Study Cohorts before and after PSM

We identified 45,219 patients with OCSCC undergoing curative surgery (39,775 without and 5445 [12.04%] with pre-existing sarcopenia) before PSM (Supplementary Table S1). Compared with the patients without pre-existing sarcopenia, those with sarcopenia were older; were predominantly women; had higher CCI scores; more likely received the diagnosis in 2015–2017; had more advanced pT and pN stages; had more poor differentiation, margin positivity, and LVI-positive tumors; and received more adjuvant concurrent chemoradiotherapy (CCRT), higher radiotherapy (RT) doses, and higher cumulative platinum doses. PSM yielded 16,294 patients (10,855 without and 5439 with sarcopenia) who were eligible for further analysis and their characteristics are summarized in Table 1. Age, sex, years of diagnosis, cancer subtypes, AJCC pathological stages, pT, pN, differentiation, surgical margin, lymphovascular invasion, adjuvant treatments, CCI scores, cigarette smoking, alcohol use, and betel nut chewing were balanced between the cohorts (all *p* > 0.05). After PSM, the crude all-cause mortality, LRR, and DM were significantly higher in the patients with sarcopenia than in those without sarcopenia (Table 1).

### 3.2. Cox Proportional Hazard Models of All-Cause Mortality

According to multivariate Cox regression analysis, pre-existing sarcopenia was a significant predictor of all-cause mortality (Table 2). Both univariate and multivariate Cox regression analyses indicated that sarcopenia was associated with poorer OS than nonsarcopenia. The HR for the univariate model was similar to that for the multivariate Cox regression analysis. Old age, male sex, advanced pT, advanced pN, differentiation grade II/III, margin positivity, LVI positivity, and CCI ≥ 1 were significantly poor prognostic factors for OS in the patients with OCSCC. In multivariate Cox regression analyses, the adjusted hazard ratio (aHRs, 95% confidence interval [CI]) of all-cause mortality for OCSCC with and without pre-existing sarcopenia was 1.14 (1.10–1.19, *p* < 0.0001). The aHRs (95% CIs) of mortality for male sex, age 50–59 years, age ≥ 60 years, pT2, pT3, pT4A, pT4B, pN1, pN2, pN3, differentiation grades II and III, margin positivity, LVI positivity, CCI ≥ 1, cigarette smoking, alcohol use, and betel nut chewing compared with female sex, age < 50 years, pT1, pN0, differentiation grade I, margin negativity, LVI negativity, CCI = 0, no cigarette smoking, no alcohol use, no betel nut chewing were 1.28 (1.20–1.39), 1.14 (1.07–1.19), 1.25 (1.19–1.33), 1.05 (1.01–1.31), 1.31 (1.05–1.63), 1.66 (1.33–2.11), 1.72 (1.39–2.17), 1.11 (1.04–1.24), 1.21 (1.05–1.41), 2.03 (1.72–2.71), 1.18 (1.12–1.23), 1.21 (1.12–1.31), 1.23 (1.18–1.33), 1.59 (1.38–1.87), 1.19 (1.13–1.26), 1.10 (1.04–1.22), 1.08 (1.03–1.23), and 1.09 (1.02–1.30), respectively.

### 3.3. Cox Proportional Hazard Models of LRR and DM

Both univariate and multivariate Cox regression analyses indicated that pre-existing sarcopenia was associated with higher risk of LRR and DM than nonsarcopenia (Table 3 and Table 4). In the multivariate Cox regression analysis, the aHRs (95% CIs) of LRR and DM for OCSCC with and without pre-existing sarcopenia were 1.07 (1.03–1.18, *p* = 0.0020) and 1.07 (1.03–1.20, *p* = 0.0148), respectively. In addition, poor prognostic factors for LRR and DM were similar with those of mortality, except old age and CCI scores. The multivariable Cox model revealed that male sex, advanced pT, advanced pN, differentiation grade II–III, margin positivity, LVI positivity, cigarette smoking use, alcohol use, and betel nut chewing use were independent poor prognostic factors for LRR and DM (Table 3 and Table 4).

### 3.4. Kaplan–Meier Curves of Overall Survival, LRR, and DM

**Figure 1:**
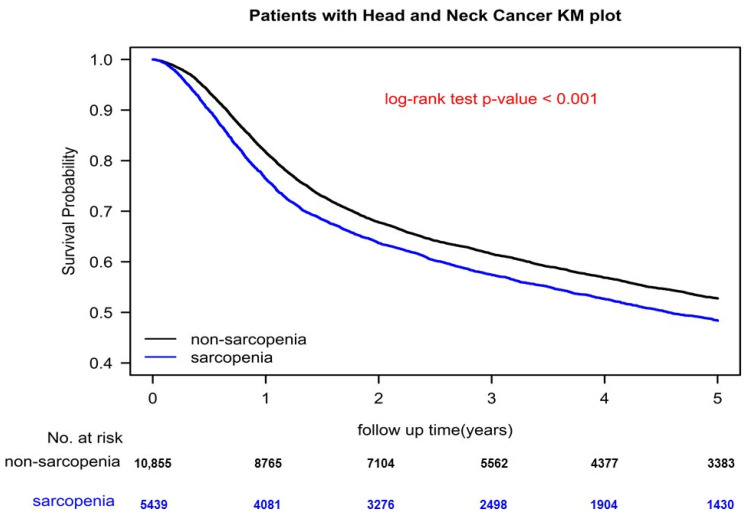
Kaplan–Meier overall survival curves for the propensity score-matched sarcopenia and nonsarcopenia groups (controls).

Figure 1 and Supplementary Figures S1 and S2 present survival curves for OS, LRR, and DM plotted using the Kaplan–Meier method for the PSM sarcopenia and nonsarcopenia OCSCC groups who underwent curative surgery. The OS curve for nonsarcopenic OCSCC was higher than that for sarcopenic OCSCC (Figure 1, *p* < 0.001). The 5-year OS was 56.03% and 48.93% for the patients with OCSCC without and with pre-existing sarcopenia, respectively. Moreover, the cumulative LRR and DM rates were significantly higher for sarcopenic OCSCC than nonsarcopenic OCSCC in the log-rank test (Supplementary Figures S1 and S2, *p* values were all <0.0001 for LRR and DM, respectively).

## 4. Discussion

Sarcopenia is an independent prognostic factor for poor survival in patients with HNC undergoing surgery, RT, or CCRT [20,42,43,44,45,46,47]. However, these studies included heterogeneous definitions of sarcopenia, inconsistent treatments for HNCs, different HNC subtypes, inhomogeneous HNC stages, very small sample sizes, and inconsistent cancer subtypes including oropharyngeal, hypopharyngeal, oral cavity, and laryngeal cancers [20,42,43,44,45,46,47]. None of these studies differentiated between sarcopenia as pre-existing or that related to cancer cachexia. Accordingly, their result that sarcopenia is a poor prognostic factor for survival outcomes might be due to cancer-related cachexia-induced sarcopenia or cancer treatment-related sarcopenia instead of pre-existing sarcopenia [20,42,43,44,45,46,47]. However, sarcopenia is different from cancer cachexia [14,16,17]. The causes of sarcopenia are multifactorial [14] and include muscle disuse, changes in endocrine function, chronic diseases, inflammation, insulin resistance, and nutritional deficiencies; many of these conditions can be detected early on and corrected through measures such as exercise or nutrition to prevent sarcopenia progression [48,49,50,51]. Therefore, we estimated the oncological outcomes of pre-existing sarcopenia in the patients with OCSCC undergoing curative surgery to determine the effect of pre-existing sarcopenia on OCSCC. To our knowledge, this is the first head-to-head PSM, largest, and longest follow-up study evaluating the effect of pre-existing sarcopenia on patients with OCSCC undergoing curative surgery. Our data indicated that pre-existing sarcopenia is an independent poor prognostic factor for mortality, LRR, and DM.

The definition of sarcopenia has been inconsistent in previous studies [20,42,43,44,45,46,47]. In patients with HNC receiving RT or CCRT, sarcopenia has been reported to be associated with poor OS and disease-free survival outcomes [42,43,44,45,47]. Only one report including patients with HNC receiving surgical excision demonstrated that sarcopenia appears to be a significant negative predictor of long-term OS in patients with HNC undergoing major surgery [43]. Stone et al. defined sarcopenia by using cross-sectional abdominal imaging performed within 45 days prior to surgery [43]. However, this definition precluded the differentiation of pre-existing sarcopenia from cancer cachexia-related sarcopenia [43]. This renders any results on the effect of sarcopenia unclear [43] and does not affect clinical practice in patients with HNC because cachexia is a well-known poor prognostic factor for OS in HNCs [52,53]. Our study is the first to present a clear definition of pre-existing sarcopenia (diagnosed ≥1 year before the diagnosis of OCSCC) in a homogenous group of patients with the same subtype of HNC (OCSCC) undergoing curative surgery. Therefore, our finding that pre-existing sarcopenia is the poor prognostic factor for OS, LRR, and DM might encourage the implementation of early screening for sarcopenia and intervention such as resistance exercise, protein supplementation, and vitamin D for patients at a high risk of OCSCC (betel nut chewing, cigarette smoking, or alcohol abuse) [48,49,50,51]. These valuable outcomes would provide references for the health government to establish health policies to correct, interrupt, or prevent the progression of pre-existing sarcopenia, particularly in the susceptible population.

Performing a randomized controlled trial (RCT) to evaluate oncological outcomes in patients with OCSCC undergoing curative surgery with and without pre-existing sarcopenia is difficult because sarcopenia cannot be treated using a tangible intervention [54]. Traditionally, striking a balance among the confounding factors of mortality in patients with OCSCC with and without sarcopenia (i.e., the case and control groups, respectively)—a main requirement of the RCT design—is impossible [54]. Although the main advantage of the PSM methodology is the more precise estimation of the covariate effect, PSM cannot control for factors not accounted for in the model. Moreover, PSM is predicated on an explicit selection bias of those who could be matched; in other words, individuals who could not be matched are not part of the scope of inference.

In the current study, our multivariable Cox regression analysis results indicated that age ≥50 years, male sex, advanced pT, advanced pN, differentiation grade II–III, margin positivity, LVI positivity, CCI ≥ 1, cigarette smoking, alcohol use, and betel nut chewing are significant poor prognostic factors for mortality—corroborating the results of previous studies (Table 2 and Figure 1) [1,2,3,4,5,6,7,8,9,10,31,55,56,57,58,59]. Moreover, male sex, advanced pT, advanced pN, differentiation grade II-III, margin positivity, LVI positivity, cigarette smoking, alcohol use, and betel nut chewing were the poor independent prognostic factors for LRR and DM in patients with OCSCC undergoing curative surgery (Table 3 and Table 4 and Supplementary Figures S1 and S2). Age > 50 years was associated with the risk of mortality in patients with HNC undergoing curative surgery, consistent with our results [3,31]. In Taiwan, male sex and high CCI scores are known poor prognostic factors for OS in patients with HNC undergoing curative surgery [3,31,59]. Our data indicated that advanced pT/pN, margin positivity, and LVI positivity are associated with an increase in all-cause mortality, LRR, and DM, consistent with previous studies and NCCN guidelines [3,30,55,56,57]. In our multivariable analysis, poor prognostic factors for oncological outcomes for patients with OCSCC undergoing curative surgery were similar to those reported in previous studies [1,2,3,4,5,6,7,8,9,10,30,31,55,56,57,58,59]. Pre-existing sarcopenia was the only independent poor prognostic factor for OS, LRR, and DM for OCSCC that was never reported in previous studies. Although cancer cachexia is a well-known poor prognostic factor for survival in HNC [52,53], ours is the first study to establish pre-existing sarcopenia as an independent prognostic factor for OCSCC.

The mechanism through which pre-existing sarcopenia serves as a poor prognostic factor for OS, LRR, and DM might be associated with multiple factors including the metabolic processes of insulin resistance and systemic inflammation [14,16,17]. Patients with sarcopenia might have systemic inflammation that reduces liver cytochrome activities and drug clearance and metabolic processes, leading to a poor therapeutic effect [60]. In addition, inflammation by sarcopenia can cause a decrease in skeletal muscle density. A decreased muscle density is related to intramuscular lipid accumulation and favored by systemic inflammation, thus leading to a vicious cycle [60]. Therefore, early intervention to break this cycle is critical in patients with sarcopenia [48,49,50,51]. According to an epidemiological study in Taiwan, the incidence of oral cancer was 123-fold higher in patients who smoked, consumed alcohol, and chewed betel quid than in abstainers [2]. Patients with sarcopenia with risk factors for OCSCC [60] are the susceptible population for poor OS. Early screening for and treatment of sarcopenia for the susceptible population might improve survival outcomes in case they develop OCSCC.

This study has several limitations. First, the cohort derived from an Asian population in Taiwan. Although no evidence indicating a significant difference in survival of OCSCC between Asian and non-Asian populations has been reported, the current results should be cautiously extrapolated to non-Asian populations. Second, this study was performed on a big database and thus it is a real challenge to rule out an ecological bias (attributed to confounding or risk factors). PSM cannot control for factors not accounted for in the model and is predicated on an explicit selection bias of the variables that were matched. Third, patients with antecedents of other cancers were excluded. The field cancerization theory is well accepted on this anatomical area, i.e., a patient with oral cancer has a higher risk to develop future aerodigestive carcinomas (and vice versa) [4,61,62]. However, the primary endpoint in the current study is the all-cause death between sarcopenia and nonsarcopenia OCSCC, OCSCC patients combined with other cancers will have higher mortality attributed to more aggressive treatments or more advanced stages on the other cancers, whatever synchronous or metachronous cancers [4,61,62]. In order to decrease the bias of all-cause death from the other cancers in the OCSCC patients, patients with antecedents of other cancers were excluded. Fourth, the diagnoses of all comorbid conditions were based on *ICD-9-CM* or *ICD-10-CM* codes in this study. Nevertheless, the Taiwan Cancer Registry Administration reviews charts and interviews of beneficiaries in the TCRD to verify the accuracy of the diagnoses, and it audits hospitals with outlier chargers or practices and subsequently heavily penalizes them if it identifies any malpractice or discrepancies. However, to obtain precise population specificity and disease occurrence data, a large-scale RCT carefully comparing patients with OCSCC with or without sarcopenia is warranted, but such RCTs may be difficult to execute.

Despite these limitations, a major strength of our study is the use of a nationwide population-based registry with detailed baseline information. The TCRD is linked with Taiwan’s National Cause of Death Database; thus, in the current study, we could perform a lifelong follow-up for most patients. Moreover, this study is the first, largest, and longest follow-up comparative cohort study to estimate the primary endpoint of OS in patients with OCSCC with and without pre-existing sarcopenia undergoing curative surgery. The covariates between the two groups were homogenous and any bias between the two groups was removed through PSM (Table 1). Considering the magnitude and statistical significance of the observed effects in the current study, the limitations are unlikely to have affected our conclusions.

## 5. Conclusions

Our results indicate that pre-existing sarcopenia is a significantly poor prognostic factor for OS, LRR, and DM in patients with OCSCC undergoing curative surgery. Individuals with a high risk of OCSCC, such as those who have a habit of betel nut chewing, alcohol, or smoking, should be screened for sarcopenia and intervention in terms of exercise and nutrition should be promoted.

## Figures and Tables

**Table 1:** Characteristics of patients with oral cavity squamous cell carcinoma with and without pre-existing sarcopenia (After propensity score matching 1:2).

	Nonsarcopenia		Sarcopenia		*p* Value
	N = 10,855		N = 5439		
	N	%	N	%	
Age (mean ± SD)	55.79 ± 10.89		55.44 ± 11.14		0.2384
Age, median (IQR), years	55.00 (48.00, 63.00)		55.00 (48.00, 63.00)		0.9929
Age groups					0.5057
<50 years	3061	28.20%	1492	27.43%	
50–60 years	3930	36.20%	1969	36.20%	
≥60 years	3864	35.60%	1978	36.37%	
Sex					0.1720
Male	9803	90.31%	4875	89.63%	
Female	1052	9.69%	564	10.37%	
Years of diagnosis					0.3349
2007–2010	2264	20.86%	1149	21.13%	
2011–2014	4612	42.49%	2246	41.29%	
2015–2017	3979	36.66%	2044	37.58%	
AJCC pathologic stage					0.9995
I	2279	21.00%	1142	21.00%	
II	1492	13.74%	747	13.73%	
III	1281	11.80%	642	11.80%	
IVA	5304	48.86%	2658	48.87%	
IVB	499	4.60%	250	4.60%	
AJCC pathologic stage T					0.9899
pT1	107	0.99%	56	1.03%	
pT2	3186	29.35%	1595	29.33%	
pT3	3270	30.12%	1637	30.10%	
pT4A	989	9.11%	497	9.14%	
pT4B	3303	30.43%	1654	30.41%	
AJCC pathologic stage N					0.9979
pN0	5117	47.14%	2572	47.29%	
pN1	1560	14.37%	779	14.32%	
pN2	3745	34.50%	1872	34.42%	
pN3	433	3.99%	216	3.97%	
Differentiation					0.9526
I	2253	20.76%	1130	20.78%	
II	6272	57,78%	3140	57.73%	
III	2330	21.46%	1169	21.49%	
Surgical margin	10,855		5439		0.9467
Negative	9078	83.63%	4539	83.45%	
Positive	1777	16.37%	900	16.55%	
Lymphovascular invasion					0.9705
No	4962	45.71%	2481	45.62%	
YES	5893	54.29%	2958	54.38%	
Adjuvant treatments					0.2968
No adjuvant	2129	19.61%	1080	19.86%	
Adjuvant RT	1452	13.38%	779	14.32%	
Adjuvant sequential CT and RT	2149	19.80%	1097	20.17%	
Adjuvant CT	322	2.97%	164	3.02%	
Adjuvant CCRT	4803	44.25%	2319	42.64%	
Adjuvant RT dose (Gy), mean	63.08 ± 15.48		63.77 ± 15.34		0.1691
Median (IQR, Q1, Q3)	66.00 (60.00, 70.00)		66.00 (60.00, 70.00)		0.1414
Adjuvant chemotherapy with cumulative platinum dose (mg), mean	542.11 ± 413.46		541.16 ± 414.90		0.9082
Median	450.00 (300.00, 650.00)		450.00 (300.00, 650.00)		0.1630
CCI scores					
Mean (SD)	0.70 ± 1.11		0.73 ± 1.13		0.2747
CCI scores					0.3813
0	7032	64.78%	3448	63.39%	
≥1	3823	35.22%	1991	36.61%	
Cigarette smoking	7590	69.92%	3794	69.76%	0.9891
Alcohol use	6299	58.03%	3144	57.80%	0.8910
Betel nut chewing	6624	61.02%	3310	60.86%	0.8872
Outcomes					
Median follow-up, y (mean ± SD)	3.87 ± 3.03		3.46 ± 2.90		<0.0001
Median follow-up, y (IQR, Q1, Q3)	3.11 (1.28, 5.81)		2.65 (1.00, 5.18)		<0.0001
All-cause mortality	10,855		5439		0.0039
No	5445	50.16%	2598	47.77%	
YES	5410	49.84%	2841	52.23%	
Metastasis					<0.0001
No	9086	83.70%	4515	83.01%	
YES	1769	16.30%	924	16.99%	
Locoregional recurrence					0.0030
No	9152	84.31%	4569	84.00%	
YES	1703	15.69%	870	16.00%	

RT, radiotherapy; CCRT, concurrent chemoradiotherapy; CCI, Charlson comorbidity index; SD, standard deviation; IQR, interquartile range; AJCC, American Joint Committee on Cancer; y, years old; N, numbers; Gy, Gray; pT, pathologic tumor stages; pN, pathologic nodal stages.

**Table 2:** Univariable and multivariable Cox proportional regression model for all-cause mortality of the propensity score-matched groups of patients with oral cavity squamous cell carcinoma with and without pre-existing sarcopenia.

	Crude HR (95% CI)		*p* Value	Adjusted HR * (95% CI)		*p* Value
Sarcopenia						
Nonsarcopenia (Ref.)	1			1		
Sarcopenia	1.18	(1.12, 1.24)	<0.0001	1.15	(1.11, 1.21)	<0.0001
Sex						
Female (Ref.)	1			1		
Male	1.36	(1.28, 1.44)	<0.0001	1.28	(1.20, 1.39)	<0.0001
Age						
<50 years (Ref.)	1			1		
50–60 years	1.06	(1.04, 1.16)	0.0430	1.14	(1.07, 1.19)	0.0021
≥60 years	1.14	(1.12, 1.22)	<0.0001	1.25	(1.19, 1.33)	<0.0001
Years of diagnosis						
2007–2010 (Ref.)	1			1		
2011–2014	0.90	(0.84, 1.06)	0.6420	0.91	(0.89, 1.08)	0.4268
2015–2017	0.77	(0.72, 1.09)	0.6664	0.83	(0.79, 1.09)	0.2332
AJCC pathologic T						
pT1 (Ref.)	1			1		
pT2	0.94	(1.04, 1.21)	0.2361	1.05	(1.01, 1.31)	0.0380
pT3	1.14	(0.92, 1.46)	0.1412	1.31	(1.05, 1.63)	0.0113
pT4A	1.64	(1.31, 2.01)	<0.0001	1.66	(1.33, 2.11)	<0.0001
pT4B	1.71	(1.37, 2.13)	<0.0001	1.72	(1.39, 2.17)	<0.0001
AJCC pathologic N						
pN0 (Ref.)	1			1		
pN1	1.51	(1.42, 1.64)	<0.0001	1.11	(1.04, 1.24)	0.0002
pN2	2.37	(2.14, 2.58)	<0.0001	1.21	(1.05, 1.41)	0.0023
pN3	3.89	(3.31, 5.03)	<0.0001	2.03	(1.72, 2.71)	<0.0001
Differentiation						
I (Ref.)	1			1		
II	1.41	(1.35, 1.43)	<0.0001	1.18	(1.12, 1.23)	<0.0001
III	1.67	(1.54, 1.80)	<0.0001	1.21	(1.12, 1.31)	<0.0001
Surgical margin						
Negative (Ref.)	1			1		
Positive	1.50	(1.42, 1.61)	<0.0001	1.23	(1.18, 1.33)	<0.0001
Lymphovascular invasion						
No	1			1		
Yes	2.16	(2.04, 2.29)	<0.0001	1.59	(1.38, 1.87)	<0.0001
Adjuvant treatments						
No adjuvant treatments (Ref.)						
Adjuvant RT	1.05	(0.82, 1.44)	0.3530	1.04	(0.92, 1.45)	0.6012
Adjuvant sequential CT and RT	1.13	(0.69, 1.84)	0.5731	1.10	(0.72, 1.82)	0.7531
Adjuvant CT	1.10	(0.67, 1.44)	0.4310	1.07	(0.79, 1.45)	0.7405
Adjuvant CCRT	1.15	(0.62, 1.91)	0.1320	1.09	(0.79, 1.31)	0.3302
CCI ≥1 (Ref. CCI = 0)	1.21	(1.18, 1.29)	<0.0001	1.19	(1.13, 1.26)	<0.0001
Cigarette smoking (Ref. no use)	1.13	(1.03, 1.34)	<0.0001	1.10	(1.04, 1.22)	<0.0001
Alcohol use (Ref. no use)	1.16	(1.08, 1.39)	<0.0001	1.08	(1.03, 1.23)	<0.0001
Betel nut chewing (Ref. no use)	1.11	(1.03, 1.41)	<0.0001	1.09	(1.02, 1.30)	<0.0001

RT, radiotherapy; CCRT, concurrent chemoradiotherapy; CCI, Charlson comorbidity index; AJCC, American Joint Committee on Cancer; y, years old; pT, pathologic tumor stages; pN, pathologic nodal stages; Ref., reference group; CI, confidence interval; HR, hazard ratio. * All the aforementioned variables in Table 2 were used in multivariate analysis.

**Table 3:** Univariable and multivariable Cox proportional regression model for locoregional recurrence of the propensity score-matched groups of patients with oral cavity squamous cell carcinoma with and without pre-existing sarcopenia.

	Crude HR (95% CI)		*p* Value	Adjusted HR (95% CI)		*p* Value
Sarcopenia						
Nonsarcopenia (Ref.)	1			1		
Sarcopenia	1.08	(1.04, 1.15)	0.0061	1.07	(1.03, 1.18)	0.0020
Sex						
Female (Ref.)	1			1		
Male	1.51	(1.37, 1.70)	<0.0001	1.46	(1.30, 1.64)	<0.0001
Age						
<50 years (Ref.)	1			1		
50–60 years	0.97	(0.90, 1.07)	0.6451	0.96	(0.90, 1.05)	0.6530
≥60 years	0.88	(0.82, 1.03)	0.3510	0.92	(0.80, 1.11)	0.2035
Years of diagnosis						
2007–2010 (Ref.)	1			1		
2011–2014	0.87	(0.50, 1.15)	0.3751	0.88	(0.52, 1.19)	0.3292
2015–2017	0.89	(0.62, 1.10)	0.2307	0.91	(0.61, 1.09)	0.2211
AJCC pathologic T						
pT1(Ref.)	1			1		
pT2	1.11	(0.86, 1.44)	0.4421	1.51	(1.15, 2.01)	0.0017
pT3	1.08	(0.83, 1.42)	0.6248	1.38	(1.05, 1.85)	0.0064
pT4A	1.03	(0.88, 1.31)	0.5462	1.21	(1.05, 1.64)	0.0110
pT4B	1.08	(0.89, 1.34)	0.6286	1.17	(1.08, 1.55)	0.0089
AJCC pathologic N						
pN0 (Ref.)	1			1		
pN1	1.13	(1.06, 1.23)	0.0012	1.12	(1.04, 1.30)	0.0017
pN2	1.04	(1.02, 1.11)	0.0269	1.17	(1.05, 1.25)	0.0002
pN3	1.13	(1.04, 1.29)	0.0006	1.21	(1.11, 1.88)	0.0008
Differentiation						
I (Ref.)	1			1		
II	1.09	(1.03, 1.16)	0.0105	1.06	(1.01, 1.14)	0.0147
III	1.13	(0.86, 1.05)	0.0962	1.12	(1.03, 1.20)	0.0188
Surgical margin						
Negative (Ref.)	1			1		
Positive	1.21	(1.18, 1.33)	<0.0001	1.20	(1.11, 1.33)	<0.0001
Lymphovascular invasion						
No						
Yes	1.08	(1.04, 1.15)	0.0022	1.30	(1.07, 1.66)	0.0011
Adjuvant treatments						
No adjuvant treatments (Ref.)						
Adjuvant RT	0.99	(0.94, 1.06)	0.7440	1.01	(0.94, 1.05)	0.7624
Adjuvant sequential CT and RT	0.97	(0.93, 1.04)	0.4545	1.00	(0.96, 1.09)	0.7827
Adjuvant CT	1.03	(0.95, 1.08)	0.7632	1.04	(0.96, 1.12)	0.2424
Adjuvant CCRT	1.11	(0.98, 1.26)	0.0922	1.09	(0.96, 1.24)	0.1145
CCI ≥1 (Ref. CCI = 0)	0.96	(0.91, 1.06)	0.3596	0.98	(0.92, 1.05)	0.8620
Cigarette smoking (Ref. no use)	1.08	(1.01, 1.22)	0.0085	1.07	(1.00, 120)	0.0431
Alcohol use (Ref. no use)	1.11	(1.03, 1.19)	0.0020	1.06	(1.01, 1.13)	0.0338
Betel nut chewing (Ref. no use)	1.31	(1.12, 1.45)	<0.0001	1.19	(1.10, 1.38)	<0.0001

RT, radiotherapy; CCRT, concurrent chemoradiotherapy; CCI, Charlson comorbidity index; AJCC, American Joint Committee on Cancer; y, years old; pT, pathologic tumor stages; pN, pathologic nodal stages; Ref., reference group; CI, confidence interval; HR, hazard ratio.

**Table 4:** Univariable and multivariable Cox proportional regression model for distant metastasis of the propensity score-matched groups of patients with oral cavity squamous cell carcinoma with and without pre-existing sarcopenia.

	Crude HR (95% CI)		*p* Value	Adjusted HR (95% CI)		*p* Value
Sarcopenia						
Nonsarcopenia (Ref.)	1			1		
Sarcopenia	1.08	(1.02, 1.15)	0.0342	1.07	(1.03, 1.20)	0.01482
Sex						
Female (Ref.)	1			1		
Male	1.72	(1.54, 1.91)	<0.0001	1.60	(1.45, 1.80)	<0.0001
Age						
<50 years (Ref.)	1			1		
50–60 years	0.93	(0.88, 1.12)	0.1793	0.98	(0.93, 1.07)	0.8381
≥60 years	0.80	(0.64, 1.09)	0.5402	0.82	(0.79, 1.07)	0.4429
Years of diagnosis						
2007–2010 (Ref.)	1			1		
2011–2014	0.98	(0.92, 1.09)	0.7552	1.03	(0.96, 1.11)	0.2075
2015–2017	1.01	(0.94, 1.12)	0.8335	1.14	(0.90, 1.19)	0.6418
AJCC pathologic T						
pT1 (Ref.)	1			1		
pT2	1.26	(0.88, 1.80)	0.1719	2.32	(1.64, 3.40)	<0.0001
pT3	1.59	(1.12, 2.28)	0.0072	2.37	(1.64, 3.34)	<0.0001
pT4A	1.71	(1.22, 2.67)	0.0001	2.44	(1.60, 3.35)	<0.0001
pT4B	1.76	(1.25, 2.49)	0.0018	2.11	(1.51, 3.33)	<0.0001
AJCC pathologic N						
pN0 (Ref.)	1			1		
pN1	1.47	(1.32, 1.65)	<0.0001	1.26	(1.14, 1.95)	<0.0001
pN2	1.80	(1.64, 1.92)	<0.0001	1.41	(1.23, 1.50)	<0.0001
pN3	2.29	(1.53, 3.42)	<0.0001	1.51	(1.22, 1.72)	<0.0001
Differentiation						
I (WD) (Ref.)	1			1		
II (moderately differentiated)	1.31	(1.21, 1.42)	<0.0001	1.08	(1.04, 1.19)	0.0110
III	1.39	(1.30, 1.58)	<0.0001	1.14	(1.08, 1.25)	0.0066
Surgical margin						
Negative (Ref.)	1			1		
Positive	1.42	(1.30, 1.56)	<0.0001	1.17	(1.07, 1.28)	0.0003
Lymphovascular invasion						
No						
Yes	1.65	(1.54, 1.79)	<0.0001	1.31	(1.10, 1.63)	0.0073
Adjuvant treatments						
No adjuvant treatments (Ref.)						
Adjuvant RT	0.96	(0.91, 1.02)	0.3243	1.02	(0.98, 1.13)	0.0755
Adjuvant sequential CT and RT	0.86	(0.78, 0.91)	<0.0001	0.94	(0.86, 1.04)	0.1688
Adjuvant CT	0.83	(0.79, 0.88)	<0.0001	0.97	(0.92, 1.05)	0.3443
Adjuvant CCRT	0.89	(0.81, 0.93)	<0.0001	1.02	(0.94, 1.09)	0.3468
CCI ≥ 1 (Ref. CCI = 0)	0.88	(0.77, 1.05)	0.1312	1.06	(0.92, 1.23)	0.2503
Cigarette smoking (Ref. no use)	1.04	(0.93, 1.20)	0.0923	1.06	(1.01, 123)	0.0207
Alcohol use (Ref. no use)	1.01	(0.91, 1.27)	0.0791	1.04	(1.00, 1.22)	0.0441
Betel nut chewing (Ref. no use)	1.07	(0.89, 1.33)	0.1201	1.04	(1.08, 1.31)	0.0363

RT, radiotherapy; CCRT, concurrent chemoradiotherapy; CCI, Charlson comorbidity index; AJCC, American Joint Committee on Cancer; y, years old; pT, pathologic tumor stages; pN, pathologic nodal stages; Ref., reference group; CI, confidence interval; HR, hazard ratio.

## Data Availability

The data sets supporting the study conclusions are included in the manuscript. We used data from the National Health Insurance Research Database and Taiwan Cancer Registry database. The authors confirm that, for approved reasons, some access restrictions apply to the data underlying the findings. The data used in this study cannot be made available in the manuscript, the supplementary files, or in a public repository due to the Personal Information Protection Act executed by Taiwan’s government, starting in 2012. Requests for data can be sent as a formal proposal to obtain approval from the ethics review committee of the appropriate governmental department in Taiwan. Specifically, links regarding contact info for which data requests may be sent to are as follows: http://nhird.nhri.org.tw/en/Data_Subsets.html#S3 and http://nhis.nhri.org.tw/point.html (accessed on 5 February 2021).

## Supplementary Materials

The following supporting information can be downloaded at: https://www.mdpi.com/article/10.3390/cancers14133246/s1, Figure S1: Kaplan–Meier overall cumulative locoregional recurrence curves for the propensity score–matched sarcopenia and nonsarcopenia groups (controls); Figure S2: Kaplan–Meier overall cumulative distant metastasis curves for the propensity score–matched sarcopenia and nonsarcopenia groups (controls); Table S1: Sensitivity analysis of washout time-intervals of one year and two years for definition of preexisting sarcopenia; Table S2: Sensitivity analysis of preexisting sarcopenia recorded by special specialties and all specialties.

## Abbreviations

RT, radiotherapy; CCRT, concurrent chemoradiotherapy; CCI, Charlson comorbidity index; SD, standard deviation; IQR, interquartile range; AJCC, American Joint Committee on Cancer; y, years old; N, numbers; Gy, Gray; pT, pathologic tumor stages; pN, pathologic nodal stages; CI, confidence interval; HR, hazard ratio; OCSCC, oral cavity squamous cell carcinoma; PSM, propensity score matching; LRR, locoregional recurrence; DM, distant metastasis; HNCs, head and neck cancers; TCRD, Taiwan Cancer Registry Database; NCCN, National Comprehensive Cancer Network; AJCC, American Joint Committee on Cancer criteria; ICD-9-CM, International Classification of Diseases, Ninth Revision, Clinical Modification; ICD-10-CM, International Classification of Diseases, Tenth Revision, Clinical Modification; CCI, Charlson comorbidity index; LVI, lymphovascular invasion; RCT, randomized controlled trial.
