# Supplementary material for: Effect of Pre-Existing Sarcopenia on Oncological Outcomes for Oral Cavity Squamous Cell Carcinoma Undergoing Curative Surgery: A Propensity Score-Matched, Nationwide, Population-Based Cohort Study
Source: Cancers (Basel). 2022 Jul 1;14(13):3246. doi: 10.3390/cancers14133246 (PMC9264926; doi:10.3390/cancers14133246)
Supplement: Supplementary file 1 [file cancers-14-03246-s001.zip › cancers-1775476-supplementary.pdf]

Supplemental Table S1. Sensitivity analysis of washout time-intervals of one year and two years for definition of preexisting sarcopenia

	One year washout						Two year washout					
	**HR (95% CI)**		***P* Value**	**aHR (95% CI)**		***P* Value**	**HR(95% CI)**		***P* Value**	**aHR (95% CI)**		***P* Value**
**Oncologic outcomes**												
**All-cause death**												
Nonsarcopenia (Ref.)	1			1			1			1		
Sarcopenia	1.18	(1.12,1.24)	<0.0001	1.15	(1.11,1.21)	<0.0001	1.15	(1.11,1.20)	<0.0001	1.11	(1.1,1.19)	<0.0001
**Locoregional recurrence**												
Nonsarcopenia (Ref.)	1			1			1			1		
Sarcopenia	1.08	(1.04,1.15)	0.0061	1.07	(1.03,1.18)	0.0020	1.05	(1.02,1.10)	0.0091	1.07	(1.03,1.09)	0.0052
**Distant metastasis**												
Nonsarcopenia (Ref.)	1			1			1			1		
Sarcopenia	1.08	(1.02,1.15)	0.0342	1.07	(1.03,1.20)	0.0148	1.06	(1.00,1.10)	0.0453	1.08	(1.01,1.19)	0.02450

RT, radiotherapy; CCRT, concurrent chemoradiotherapy; CCI, Charlson comorbidity index; AJCC, American Joint Committee on Cancer; y, years old; pT, pathologic tumor stages; pN, pathologic nodal stages; Ref., reference group; CI, confidence interval; HR, hazard ratio.

*All the aforementioned variables in Table 2 were used in multivariate analysis.

Supplemental Table S2. Sensitivity analysis of preexisting sarcopenia recorded by special specialties and all specialties

	Recorded by special specialties						Recorded by all specialties					
	**HR (95% CI)**		***P* Value**	**aHR (95% CI)**		***P* Value**	**HR(95% CI)**		***P* Value**	**aHR (95% CI)**		***P* Value**
**Oncologic outcomes**												
**All-cause death**												
Nonsarcopenia (Ref.)	1			1			1			1		
Sarcopenia	1.18	(1.12,1.24)	<0.0001	1.15	(1.11,1.21)	<0.0001	1.18	(1.14,1.23)	<0.0001	1.15	(1.11,1.21)	<0.0001
**Locoregional recurrence**												
Nonsarcopenia (Ref.)	1			1			1			1		
Sarcopenia	1.08	(1.04,1.15)	0.0061	1.07	(1.03,1.18)	0.0020	1.11	(1.06,1.19)	<0.0001	1.11	(1.07,1.23)	<0.0001
**Distant metastasis**												

Nonsarcopenia (Ref.)	1			1			1			1		
Sarcopenia	1.08	(1.02,1.15)	0.0342	1.07	(1.03,1.20)	0.0148	1.08	(1.03,1.16)	0.0003	1.11	(1.04,1.26)	0.0002

RT, radiotherapy; CCRT, concurrent chemoradiotherapy; CCI, Charlson comorbidity index; AJCC, American Joint Committee on Cancer; y, years old; pT, pathologic tumor stages; pN, pathologic nodal stages; Ref., reference group; CI, confidence interval; HR, hazard ratio.
*All the aforementioned variables in Table 2 were used in multivariate analysis.

Supplemental Figure S1. Kaplan–Meier overall cumulative locoregional recurrence curves for the propensity score–matched sarcopenia and nonsarcopenia groups (controls).

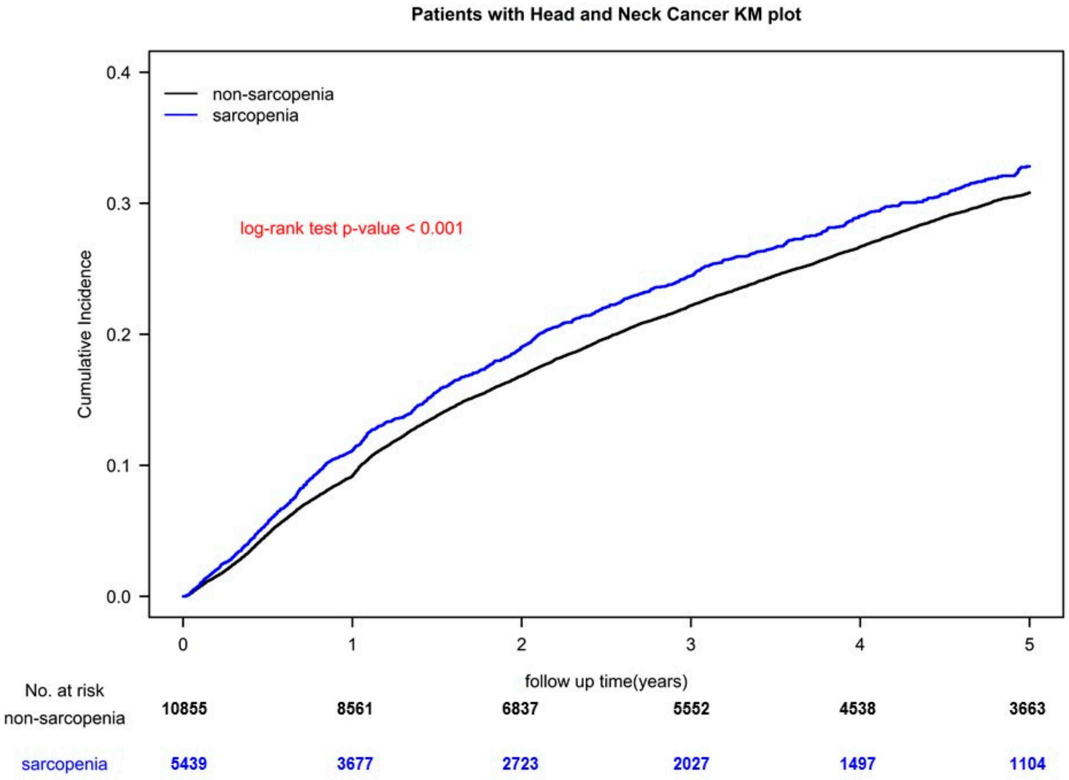

Supplemental Figure S2. Kaplan–Meier overall cumulative distant metastasis curves for the propensity score–matched sarcopenia and nonsarcopenia groups (controls).

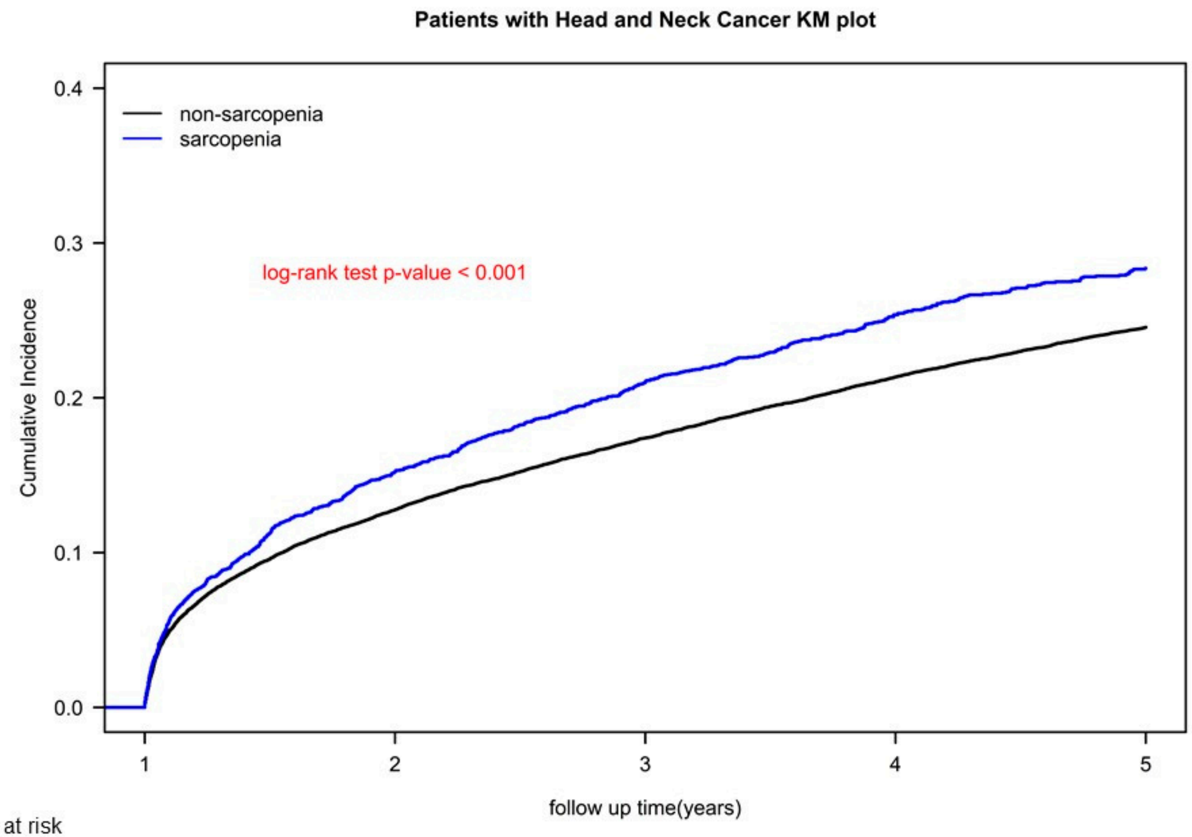

No. at risk					
non-sarcopenia	**10855**	**8627**	**7131**	**5889**	**4838**
sarcopenia	**5439**	**3945**	**2986**	**2239**	**1679**
